# Climate Determinants of Keratoconus: Insights From a Systematic Review of Prevalence

**DOI:** 10.1167/iovs.66.2.30

**Published:** 2025-02-11

**Authors:** Hasan Shabani, Job De Ridder, Mohammad Ali Asaad, Wichor M. Bramer, Magda A. Meester-Smoor, Annette A. J. M. Geerards, Caroline C. W. Klaver, Wishal D. Ramdas, Bart T. H. van Dooren

**Affiliations:** 1 Department of Ophthalmology, Erasmus University Medical Center, Rotterdam, the Netherlands; 2 Department of Epidemiology, Erasmus University Medical Center, Rotterdam, the Netherlands; 3 Cornea Center, The Rotterdam Eye Hospital, Rotterdam, the Netherlands; 4 Experimental Eye Research Institute, University Eye Hospital, Ruhr-University Bochum, Bochum, Germany; 5 Medical Library, Erasmus University Medical Center, Rotterdam, the Netherlands; 6 Department of Ophthalmology, Radboud University Medical Center, Nijmegen, the Netherlands; 7 Institute of Molecular and Clinical Ophthalmology, University of Basel, Basel, Switzerland; 8 Department of Ophthalmology, Amphia Hospital, Breda, the Netherlands

**Keywords:** ultraviolet rays, humidity, geographic locations, epidemiology, incidence, keratoconus, cornea

## Abstract

**Purpose:**

The reported prevalence of keratoconus varies widely worldwide, but the causes of this variation are not well understood. We therefore aimed to explore the potential impact of local climate variables on keratoconus prevalence.

**Methods:**

The worldwide prevalence of clinical keratoconus in the general population was systematically reviewed. In each eligible prevalence area, four climate variables deemed possibly relevant to keratoconus were assessed: daily maximum temperature, relative humidity, ultraviolet radiation, and wind speed. Climate variables were calculated using worldwide gridded climate datasets from the European Center of Medium-Range Weather Forecasts. Population density weighting was applied to enhance exposure accuracy. The average of each climate variable was calculated over the 10 years preceding data collection of each study. The potential impact of those climate variables was investigated using multiple linear regression adjusted for the gross domestic product per capita (based on purchasing power parity) with the natural logarithm of prevalence as the outcome variable.

**Results:**

Sixteen eligible studies were identified. After filtering to retain one prevalence estimate per region, 11 studies including datapoints from 61 areas were analyzed. The median (interquartile range) prevalence of keratoconus was 0.10% (0.07%–0.19%). Multiple regression revealed a significant negative association between humidity and keratoconus prevalence (β = −0.03; 95% confidence interval, −0.06 to −0.01; *P* = 0.004). In contrast, the other analyzed climate variables were not significantly associated with keratoconus prevalence.

**Conclusions:**

Using global gridded climate maps, we observed a significant and biologically plausible link between low humidity and keratoconus. This suggests that humidification could benefit patients and at-risk groups.

Keratoconus is characterized by thinning and steepening of the cornea. Keratoconus can lead to irregular astigmatism and decreased visual acuity, and its etiology is not completely understood yet. The reported keratoconus prevalence estimates vary greatly worldwide, ranging between 2 and 47,900 per million. This variation seems to be related to geographic location. For example, the reported prevalence of keratoconus in the Middle East seems substantially higher than in Europe or East Asia. Interethnic genetic differences are likely to play a role in this variation. Additionally, study design differences and definition heterogeneity can greatly contribute to variations in the reported prevalence.^1^^,^^2^ Another perspective that has not been properly investigated thus far is that some climate factors might influence the risk of acquiring keratoconus.

The connection between climate conditions and corneal health is biologically plausible. After all, the cornea lies very close to the ambient environment during waking hours, protected only by a thin tear film. A connection between keratoconus and ultraviolet (UV) exposure was previously proposed. Although UVA plays a known role in crosslinking, UVB might damage the cornea.^3^ Interestingly, there were some mixed findings regarding the potential benefits of using sunglasses for keratoconus patients.^4^ Previous research has also demonstrated an interesting link between being raised in a dry and cool area and having a thinner cornea.^5^ Additionally, elevated temperature, increased windspeed, and low humidity can lead to a higher risk of having dry eye disease (DED) and eczema.^6^^,^^7^ These two conditions are known risk factors for keratoconus.^1^^,^^8^

Although the previously mentioned studies suggest a possible link between climate and keratoconus, this correlation has not been assessed on a global scale so far. We therefore aimed to systematically review the prevalence of keratoconus in the general population and to investigate the associations between the reported prevalence estimates and four selected climate factors: daily maximum temperature, relative humidity, UV exposure, and windspeed. Such an analysis might provide novel insights into the pathophysiologic mechanisms underlying keratoconus and aid management efforts.

## Methods

The study involved four consecutive parts: a systematic review of the prevalence of keratoconus, filtering studies for climate analysis, collecting climate averages and economic indicators from the respective areas of the selected studies, and modeling the relationship between climate factors and the prevalence of keratoconus. Supplementary Figure S1 gives a visual summary of the methods.

### Systematic Review

Our aim was to find reliable and comparable estimates of the prevalence of clinical keratoconus around the world. This systematic review was conducted and reported in accordance with the 2020 Preferred Reporting Items for Systematic Reviews and Meta-Analyses (PRISMA) recommendations.^9^ We only considered registry-based studies that provide a reasonable estimate of the prevalence (or incidence) of clinical keratoconus in the general population of each region. Registry-based studies, such as those utilizing insurance or hospital databases, typically only count patients who need medical attention as cases. In contrast, keratoconus screening studies may also include milder and subclinical cases, leading to higher prevalence estimates.^1^^,^^2^ Additionally, the definitions used in screening studies vary widely, with a recent analysis showing poor agreement among published screening definitions (median Cohen's κ = 0.20).^10^ This makes it challenging to perform statistical corrections for comparability. For these reasons, we excluded screening studies. Additionally, studies focused on specific subpopulations, such as children or patients from refractive surgery clinics, were also excluded.

The search queries were developed by a medical informatics specialist (WMB) and covered six databases (MEDLINE Ovid, embase.com, Web of Science Core Collection, LILACS, Scielo, and Global Index Medicus). The search combined terms for keratoconus or conic cornea and prevalence or epidemiology. The full queries and coverage periods of each database are detailed in Supplementary Methods S1. The screening of titles and abstract was performed in EndNote 20.6 (Clarivate, Philadelphia, PA, USA) using the methodology suggested by Bramer et al.^11^^,^^12^ In short, two reviewers (JDR and MAA) independently screened and filtered the studies. In case of disagreement, a third assessor (HS) was consulted.

Data were collected from the included studies by two reviewers (HS and JDR). The following data points were extracted from each included study: first author name, publication year, point prevalence, study design, method of determining prevalence, geographical area(s), and available population characteristics (age, sex, and race/ethnicity). If point prevalence was not reported, incidence (new cases per 100.000 person-years) was collected instead. For studies that draw data from a publicly accessible database, the prevalence estimates were directly taken from that database. If only incidence was reported in the study, we estimated prevalence using a well-known epidemiologic formula (prevalence ≈ incidence × average disease duration) as explained in detail in Supplementary Methods S2. A relevant geographical exposure area was defined for each prevalence estimate. For studies using data from healthcare systems or insurance providers, this area was equivalent to the shape of the pertinent administrative area (i.e., country, state, or province). In studies using data from a health facility, a 30-km radius around the facility was used. This distance was arbitrarily chosen, as it encompasses the typical catchment area of most health facilities.^13^^,^^14^ Given the coarse resolution of the climate data used (as discussed later), defining a smaller reference area offered no significant benefit.

Using the tool provided by Hoy et al.,^15^ the risk of bias for each included study was assessed. This assessment was independently carried out by two reviewers (HS and JDR). In case of discrepancies, differences were discussed until a consensus was reached. To assess interobserver agreement, the quadratic kappa was calculated.

### Study Selection for the Climate Analysis

To ensure that the independent observations assumption of linear regression held, studies were filtered to keep one datapoint per area. We favored studies that assessed keratoconus frequency in more sub-areas. If equal numbers of areas were assessed, we chose studies that directly reported prevalence (not incidence). In case of a tie, we selected the more recent study.

### Climate Data

Daily maximum temperature, relative humidity, UV exposure, and windspeed were assessed. The average of each climate variable was calculated for each studied region over the 10 years preceding the start of data collection in each study. This period was chosen to smooth climate fluctuations. Climate variables were retrieved from reanalysis datasets, which use historical weather observations and meteorological modeling to provide a spatially continuous estimate of the climate for the entire globe. Such datasets can provide accurate weather estimates even in areas with few weather stations. More specifically, we used the ERA5 dataset and its derivatives.^16^ The ERA5 datasets are curated by the European Center of Medium-Range Weather Forecasts (ECMWF). They combine data from a number of sources, including local weather stations and satellites. The datasets cover the entire Earth from as early as 1940 (depending on the specific dataset) onward at a resolution of 0.25° × 0.25° (approximately 27 × 27 km at the equator).^16^

The spatial format of the ERA5 datasets facilitates data manipulation. Using gridded climate datasets, one can extract the variables of interest over a period of interest (e.g., 10 years) and average it over time. This results in a global map of 10-year averages at each coordinate. It is then possible to overlay a shape (e.g., the outline of a province) and calculate the spatial average of the data within this shape. This gives a coarse estimate of the population's exposure to the studied climate variables. However, such an estimate might not be that accurate in areas with diverse climates and unevenly distributed inhabitants. An illustrative example of this is given in Supplementary Figure S2, which contrasts the population distribution in Nevada with the distribution of daily maximum temperature. The two seem to vary widely across the state; therefore, to capture the real exposure, we calculated the population-weighted average of each climate variable in each studied area. Supplementary Methods S3 delves deeper into the methods utilized to extract and process climate variables.

### Gross Domestic Product

The reported prevalence of keratoconus may be influenced by regional differences in healthcare standards. To account for this, we incorporated the gross domestic product adjusted for purchasing power parity (GDP-PPP) per capita as a general measure of prosperity. Data for GDP-PPP per capita were collected for the final year of data collection for each study. For the United States, we calculated state-level GDP-PPP per capita by adjusting state-level real GDP (in chained 2017 dollars) using the regional price parity (RPP) index and dividing the result by the population estimate for the corresponding year. State-level GDP and RPP data were taken from the US Bureau of Economic Analysis,^17^ and population estimates were taken from the US Census Bureau.^18^ For regions outside the United States, country-level GDP-PPP per capita estimates were directly obtained from the International Monetary Fund database using 2017 international dollars.^19^ Relying on the 2017 dollar as a currency for all GDP data points helped account for changes in the real purchasing power of the dollar across time.

### Statistical Analysis

The mean, standard deviation, and quartiles of prevalence; GDP-PPP per capita; and climate data were calculated and presented. Because the denominator was not available for most of the included prevalence estimates, a meta-analysis was not performed. After exploring climate and prevalence data, a multivariable linear regression model was fitted using GDP-PPP per capita and the four studied climate factors as predictors, with the natural logarithm of prevalence as an outcome variable. Prevalence was transformed to ensure the normality of the residuals. A simplified parsimonious model was consequently fitted including only the significant predictors from the full model. A few prevalence points were not directly reported but derived from the reported incidence. To ensure the robustness of the results, we conducted a sensitivity analysis excluding the prevalence estimates we derived from incidence. Stratifying for race was not possible due to limited reporting in most studies. Nevertheless, we performed another sensitivity analysis to see if the observed effects were merely artifacts of the underlying interethnic differences. This sensitivity analysis involved only those areas where the disease frequency was reported among Europeans only or areas where the majority (>75%) of inhabitants are of European ancestry according to official census data. *P* ≤ 0.05 was considered statistically significant. Statistical tests were performed using R 4.1.1 (R Foundation for Statistical Computing, Vienna, Austria)^20^ and RStudio 1.4.1717 (RStudio, Boston, MA, USA).^21^

## Results

### Systematic Review

A total of 3709 unique records were screened. Supplementary Figure S3 summarizes the filtering process. Of the 16 eligible studies, five relied on the registry of a health facility with a well-defined catchment area,^22^^–^^26^ and the remaining studies relied on insurance or healthcare system registries.^27^^–^^37^ Seven of the included studies had a moderate risk of bias, but the rest had a low risk of bias (Table 1). There was a good agreement between the risk of bias raters (quadratic κ = 0.71; *P* = 2e-19).

**Table 1.** Summary of Eligible Studies.

Study	Selected for Climate Analysis?	Study Area	Prevalence (%)	Risk of Bias
Bak-Nielsen et al. (2019)^27^	Yes	Denmark	0.044	Low
Georgiou et al. (2004)^22^	Yes	Yorkshire (United Kingdom)	0.342^*^	Intermediate
Godefrooij et al. (2017)^28^	Yes	The Netherlands	0.265	Low
Hwang et al. (2018)^29^	Yes	South Korea	0.037	Low
Ihalainen (1986)^23^	Yes	Oulu hospital district (Finland)	0.029	Low
Kennedy et al. (1986)^24^	No	Olmsted County, MN (United States)	0.055	Intermediate
Kristianslund et al. (2021)^31^	Yes	Norway	0.192	Low
Mejia-Salgado et al. (2023)^37^	Yes	Colombia	0.081^*^	Low
Moon et al. (2020)^30^	No	South Korea	NA	Intermediate
Munir et al. (2021)^32^	Yes	United States	0.150	Intermediate
Ng et al. (2023)^33^	Yes	Taiwan	0.068^*^	Low
Nielsen et al. (2007)^34^	No	Denmark	0.086	Low
Pearson et al. (2000)^26^	Yes	Leicestershire (United Kingdom)	0.038	Intermediate
Reeves et al. (2009)^35^	No	United States	0.016%	Intermediate
Singh et al. (2023)^36^	No	United States	0.040	Intermediate
Ziaei et al. (2012)^25^	Yes	Province of Yazd (Iran)	0.669^*^	Low

* Prevalence was not directly reported but instead was estimated from incidence. The study of Munir et al.^32^ contains state-level data, as well. NA, not reported and not estimated.

### Study Selection for the Climate Analysis

Studies from the same country were filtered based on the criteria mentioned in the Methods section. Five studies were excluded, and 11 studies were carried forward to the climate analysis (Table 1).

### Climate Data

The 11 studies that were selected for the climate analysis reported the disease frequency in 61 different regions distributed across four different continents: Asia, Europe, and North and South America. Table 2 and Supplementary Figure S4 display the reported or estimated prevalence, as well as the distribution of the 10-year average population-weighted climate data for each studied area.

**Table 2.** Descriptive Statistics of Study Variables

Variable	*N* (Missing)	Mean	SD	25th Percentile	Median	75th Percentile
Disease prevalence (percentage)	61 (0)	0.14	0.12	0.07	0.10	0.19
Disease prevalence (log-transformed)	61 (0)	−6.82	0.71	−7.26	−6.91	−6.27
Daily maximum temperature (°C)^*^	61 (0)	17.63	5.30	13.89	17.06	22.33
Relative humidity (percentage)^*^	61 (0)	67.31	10.83	65.60	68.94	71.83
UV exposure (MJ/m^2^)^*^	61 (0)	1.75	0.34	1.63	1.73	1.92
Wind speed (m/s)^*^	61 (0)	2.73	0.75	2.27	2.63	3.18
GDP-PPP per capita (thousands of USD)	61 (0)	57.76	19.35	48.09	56.32	63.62

* Population-weighted average.

### Climate, GDP, and Keratoconus Prevalence

Table 3 summarizes the results of the fitted linear regression models. When adjusting for GDP-PPP per capita and other climate variables, a significant negative association was found between relative humidity and the natural logarithm of prevalence (β = −0.03; 95% confidence interval [CI], −0.06 to −0.01; *P* = 0.004). This means that prevalence increased about 3% (of its initial value) with every 1% decrease in relative humidity if the other climate variables and the GDP-PPP per capita were held constant. The other assessed climate factors were not significantly associated with the prevalence of keratoconus, but GDP-PPP per capita showed a significant association with the reported prevalence (β = 0.01; 95% CI, 0.00−0.02; *P* = 0.045). A parsimonious model including only GDP-PPP per capita and relative humidity as predictors showed comparable results (Table 3). When excluding studies in which prevalence was not directly reported, the association between humidity and keratoconus remained significant (β = −0.04; 95% CI, −0.05 to −0.03; *P* < 0.001). The same was also true when analyzing only the areas with >75% European descendants (β = −0.05; 95% CI, −0.08 to −0.01; *P* = 0.011).

**Table 3.** Multivariable Linear Regression Results

Study Variable	β (95% CI)	*P*
**Model I (full model including all areas and all study variables)**		
Constant	−5.49 (−7.99, −2.99)	<0.001
Daily maximum temperature (°C)^*^	−0.01 (−0.06, 0.05)	0.835
Relative humidity (percentage)^*^	−0.03 (−0.06, −0.01)	0.004^†^
UV exposure (MJ/m^2^)^*^	0.28 (−0.73, 1.29)	0.580
Wind speed (m/s)^*^	0.01 (−0.23, 0.26)	0.922
GDP-PPP per capita (thousands of USD)	0.01 (0.00, 0.02)	0.045^†^
**Model II (parsimonious model including only the significant predictors from Model I)**		
Constant	−4.74 (−5.74, −3.74)	<0.001
Relative humidity (percentage)^*^	−0.04 (−0.05, −0.02)	<0.001^†^
GDP-PPP per capita (thousands of USD)	0.01 (0.00, 0.02)	0.041^†^
**Sensitivity Analysis I (including only studies where prevalence was directly reported in the article)**		
Constant	−4.91 (−5.98, −3.84)	<0.001
Relative humidity (percentage)^*^	−0.04 (−0.05, −0.03)	<0.001^†^
GDP-PPP per capita (thousands of USD)	0.01 (0.01, 0.02)	0.001^†^
**Sensitivity Analysis II (including only estimates from a population of >75% European descendants)**		
Constant	−4.97 (−7.92, −2.02)	0.002
Relative humidity (percentage)^*^	−0.05 (−0.08, −0.01)	0.011^†^
GDP-PPP per capita (thousands of USD)	0.02 (0.00, 0.05)	0.053

There were 61 observations in Models I and II, 57 observations in Sensitivity Analysis I, and 24 observations in Sensitivity Analysis II.

* Population-weighted average.

† Statistically significant.

## Discussion

In this systematic review, the worldwide prevalence of clinical keratoconus ranged between 0.02% and 0.67%. After considering different climate factors, low humidity emerged as an unexplored and potentially modifiable risk factor for keratoconus. Conversely, daily maximum temperature, UV exposure, and windspeed were not significantly associated with the prevalence of keratoconus.

### Keratoconus and Low Humidity

**Figure 1.**
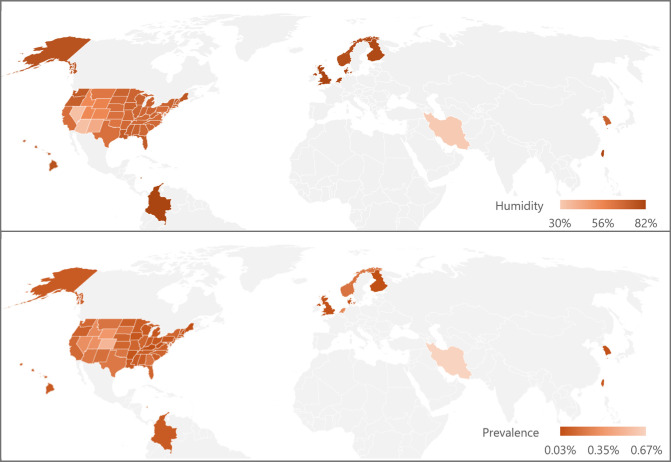
Distribution of humidity (*top*) and prevalence (*bottom*) across the included study regions. The maps were created using Microsoft Excel 365. The entire state or country was shaded uniformly, even when data were derived from a smaller subregion. Hawaii was manually enlarged and repositioned to improve visibility. In the United Kingdom, two prevalence estimates were available; this graph displays data from the study that directly reported prevalence values.

**Figure 2.**
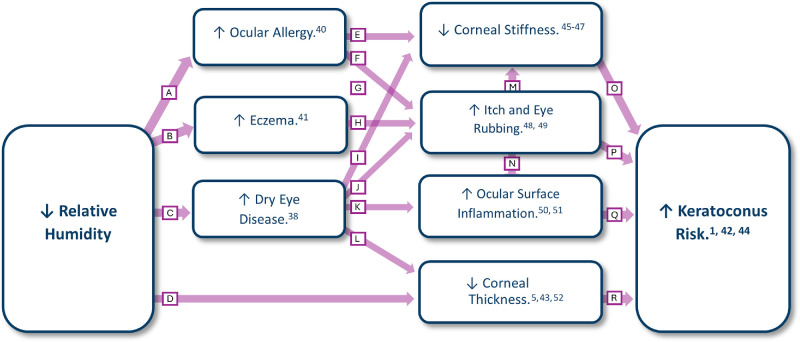
Suggested pathways for the relationship between low humidity and keratoconus. The *letter* on each *arrow* describes relevant findings from the cited studies. (**A**) Allergic conjunctivitis visits were significantly less when relative humidity increased (odds ratio = 0.998; *P* < 0.001) based on a multiple logistic regression model. (**B**, **F**–**H**, **J**, **K**, **N**, **P**, **Q**) Descriptive/review articles. (**C**) Participants who lived in places with <70% relative humidity showed a higher prevalence of DED than others (17.7% vs. 13.6%; *P* < 0.01) based on a log-linear model adjusted for age and sex. (**D**) Average relative humidity mean in the last 40 years was significantly correlated with minimum corneal thickness (Pearson correlation *r* = 0.564; *P* = 0.010). (**E**) First applanation (A1) length and the stiffness parameter at first applanation (SP-A1) were significantly lower in the vernal keratoconjunctivitis group (*P* = 0.002 and *P* = 0.04, respectively) based on the independent samples *t*-test. (**I**) In DED patients, conjunctival staining scores were significantly associated with second applanation velocity (*P* = 0.04), and corneal staining scores were significantly associated with the second applanation length (P = 0.01). This was assessed using a mixed-effects linear regression model adjusted for spherical equivalent refraction, intraocular pressure and central corneal thickness. (**L**) Dry eye patients had significantly lower central corneal thickness (CCT) compared to age- and gender-matched healthy controls (mean CCT = 537 vs. 561 µm; *P* < 0.01) based on the paired *t*-test. (**M**) After 1 minute of eye rubbing, smaller SP-A1 (*P* < 0.001), higher deformation and deflection amplitudes (*P* < 0.001 and *P* = 0.012, respectively), higher peak distances (*P* < 0.001), earlier A1 times (*P* < 0.001), faster velocities (*P* < 0.001), and lower maximum inverse radii (*P* = 0.004) were observed. These results are from the paired *t*-tests. (**O**) Inverse-variance weighted Mendelian randomization (IVW-MR) suggested that the corneal resistance factor has a negative causal relationship with keratoconus (β = −1.37 ± 0.086). (**R**) Similarly, IVW-MR suggested that CCT has a negative casual relationship with keratoconus (β = −0.027 ± 0.0021).

Figure 1 shows how relative humidity and keratoconus prevalence compared in the studied regions. To the best of our knowledge, the relationship between keratoconus and low humidity has not been previously described or explored. As Figure 2 suggests, DED might be a mediator in this relationship, as low humidity is directly associated with DED,^38^ and a history of DED is a known risk factor for keratoconus.^8^^,^^39^ Other possible mediators of the association between keratoconus and low humidity include eczema and ocular allergy. These two conditions seem more frequent under low humidity conditions^40^^,^^41^ and are known keratoconus risk factors.^1^^,^^42^ Finally, low humidity is associated with less corneal thickness,^5^^,^^43^ a key feature of keratoconus that might even play a causal role.^44^ Given the collective evidence from these studies and others, the relationship between low humidity and keratoconus seems fairly reasonable from a biological point of view. These hypothesized mediators may directly or indirectly contribute to a higher risk of developing keratoconus in a genetically predisposed individual, as Figure 2 shows.^45^^–^^52^ Additional investigations are necessary to verify the assumed directions of causality and to check for other possible pathways.

### Keratoconus and UV Radiation

Theoretically, UV radiation might have two opposing effects on the cornea. Excessive UVB is known to damage corneal tissue due to oxidative stress, whereas UVA might enhance crosslinking and reinforce the cornea.^3^ It was previously suggested that UV plays a role in the development of keratoconus.^53^ However, UV exposure was not significant in our analysis when adjusting for other climate variables. A previous study suggested that high altitude might be associated with a higher incidence or severity of keratoconus.^54^ The proposed explanation was that UV exposure increases with altitude.^54^ However, this correlation might also be explained by the fact that relative humidity decreases linearly by an average of 4% for every 1000-meter increase in altitude.^55^

### Prevalence and Economic Prosperity

All of the countries covered in this analysis had very good (>90%) healthcare coverage.^33^^,^^56^^,^^57^ Nevertheless, the multivariable analysis showed that GDP-PPP per capita was positively associated with the reported prevalence of keratoconus, highlighting possible discrepancies in eyecare standards and/or accessibility.

### Strengths and Limitations

A great advantage of this analysis is its global nature, allowing the comparison of diverse areas with diverse climate characteristics. The effects of climate variables on ectasia were not compared or modeled together before. Given the scope of this review, our search was not limited by language, and international databases such as LILACS were also consulted. This study also introduces a relatively new design in the field of ophthalmology which relies on gridded global climate datasets to investigate the relationship between meteorological variables and health. State-of-the-art datasets and weighting approaches were used to measure climate factors. Nevertheless, the effective population exposure levels remain challenging to estimate. Correcting, for example, for the hours spent outside and for the use of humidifiers, sunscreen, and air conditioning would have improved accuracy. However, we were not able to find reliable sources that provide such data on a global level. The lack of homogeneous data from the relevant study years also prevented us from including air pollution variables. Pollutants are a potential risk factor for keratoconus.^58^ Exploring their effects and interactions with other climate factors in relation to keratoconus risk would be valuable; however, pollution can significantly vary from year to year, and the exposure to pollutants can vary largely within the same metropolitan area (e.g., urban vs. suburban areas). This variation underscores the need for consistent, high-quality, and population-weighted pollution data to understand their real risk.

Adjusting for ancestry was not possible due to limited and inconsistent reporting in the included studies. However, the sensitivity analysis among areas where >75% of inhabitants were of European ancestry supported the results and conclusions of this study. One caveat about the sensitivity analysis is that census data in the United States classify some Middle Eastern ethnicities in the same category (White alone) as Europeans. Keratoconus might be more prevalent in these groups than in Europeans.^1^ However, given the lack of a better alternative and the relatively small percentages of these ethnic minorities in the United States, the official census percentages of (White alone) inhabitants were used. The statistical analysis performed in this study assumed that the reported prevalence estimates are homogeneous and comparable. To ensure this, we opted to exclude screening studies as explained in the Methods section. We also adjusted the analysis for GDP-PPP per capita to account for differences in eyecare standards. However, the GDP-PPP per capita is a proxy (i.e., indirect) measure and may not fully capture these differences. Four out of 61 prevalence estimates included in our analysis were not directly reported, but we derived them from incidence as explained in Supplementary Methods S2. Table 3 shows that the results and conclusions were robust to the exclusion of these data points. Finally, two of the included studies were based on data from large private insurance providers. It was difficult to verify if prevalence among these policyholders gives an accurate representation of the prevalence in the general population.

### Future Directions

Given the geographic and biological plausibility of a link between low humidity and keratoconus, future studies could investigate whether this association is replicable in vivo or on a local scale. Local studies can use climate data of higher resolution and incorporate a more standardized disease definition. Moreover, local studies facilitate correcting for more potential confounders such as ethnicity and the number of hours spent outside. Furthermore, a better understanding of the possible role of pollutants in keratoconus can be achieved by combining high-resolution (e.g., postal code level) data for both pollution exposure and disease incidence. Because climate (i.e., the general prevailing weather patterns) was the main topic of this analysis, a 10-year reference period was used. Another interesting research direction is to explore the possible effects of short-term weather fluctuations on keratoconus risk using incidence data. Future analyses may also incorporate different modeling techniques such as hierarchical modeling.

On a practical level, clinical studies should explore the potential preventive and/or stabilizing applications of corneal humidification methods that are not commonly prescribed in the context of keratoconus. For example, humidifiers may help by increasing indoor humidity, warm compresses may support the tear-film stability, and protective eyewear may safeguard the cornea in arid climates. These simple interventions are characterized by their low cost, minimal side effects, and ease of application, making them particularly promising for real-world applications. Finally, this novel approach, utilizing global analysis of gridded climate datasets, can be extended to explore the environmental underpinnings of other ocular diseases, as well.

## Supplementary Material

Supplement 1
