# Supplementary material for: Climate Determinants of Keratoconus: Insights From a Systematic Review of Prevalence
Source: Invest Ophthalmol Vis Sci. 2025 Feb 11;66(2):30. doi: 10.1167/iovs.66.2.30 (PMC11817973; doi:10.1167/iovs.66.2.30)
Supplement: Supplement 1 [file iovs-66-2-30_s001.pdf]

**SUPPLEMENTARY FIGURE S1.** Visual Summary of The Study Design

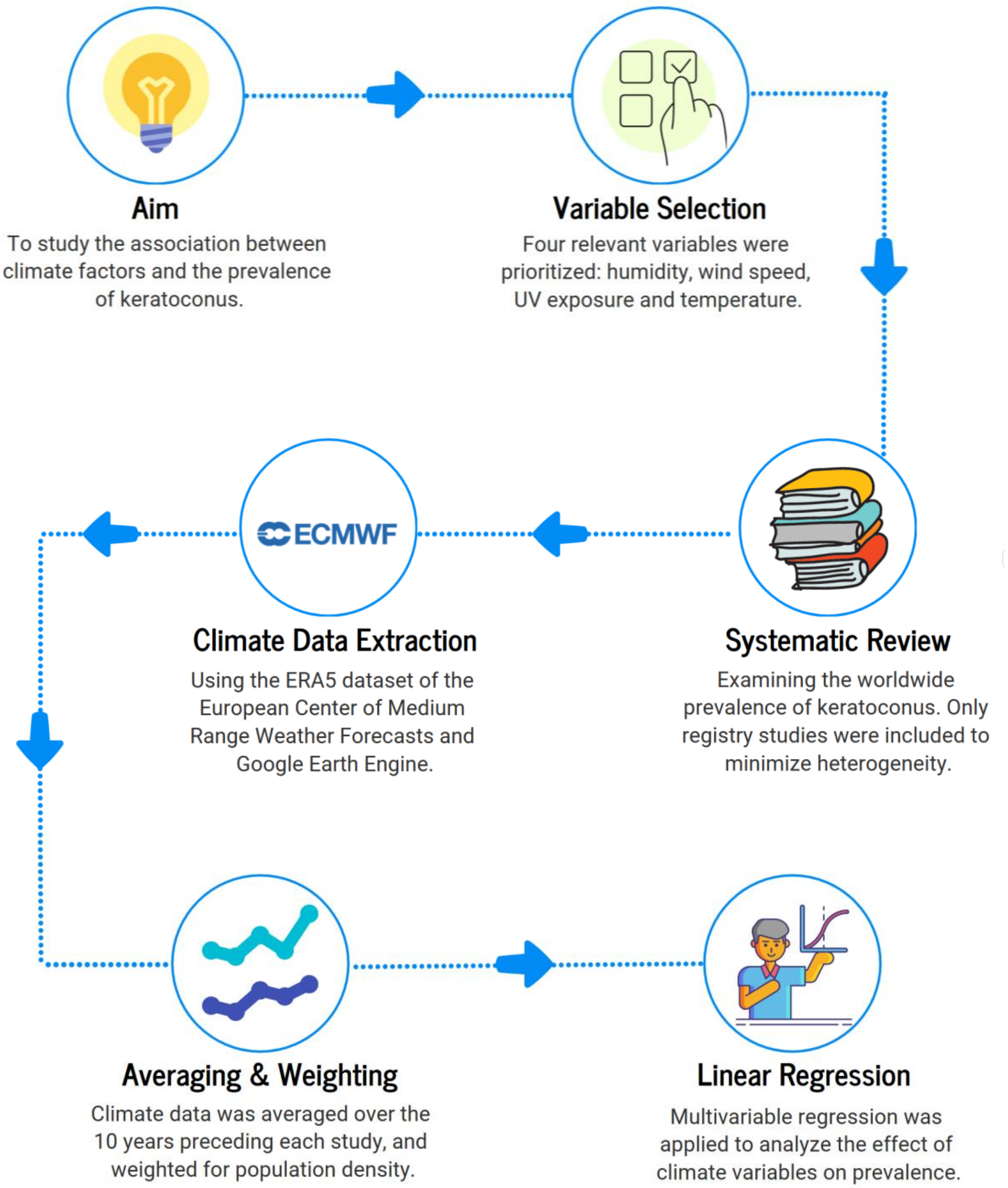

**SUPPLEMENTARY FIGURE S2.** An Illustrative Example Showing the Need for Population Weighting. Left: Population Distribution of Nevada in 2010; Right: Average Daily Maximum Temperature of Nevada in 2010.

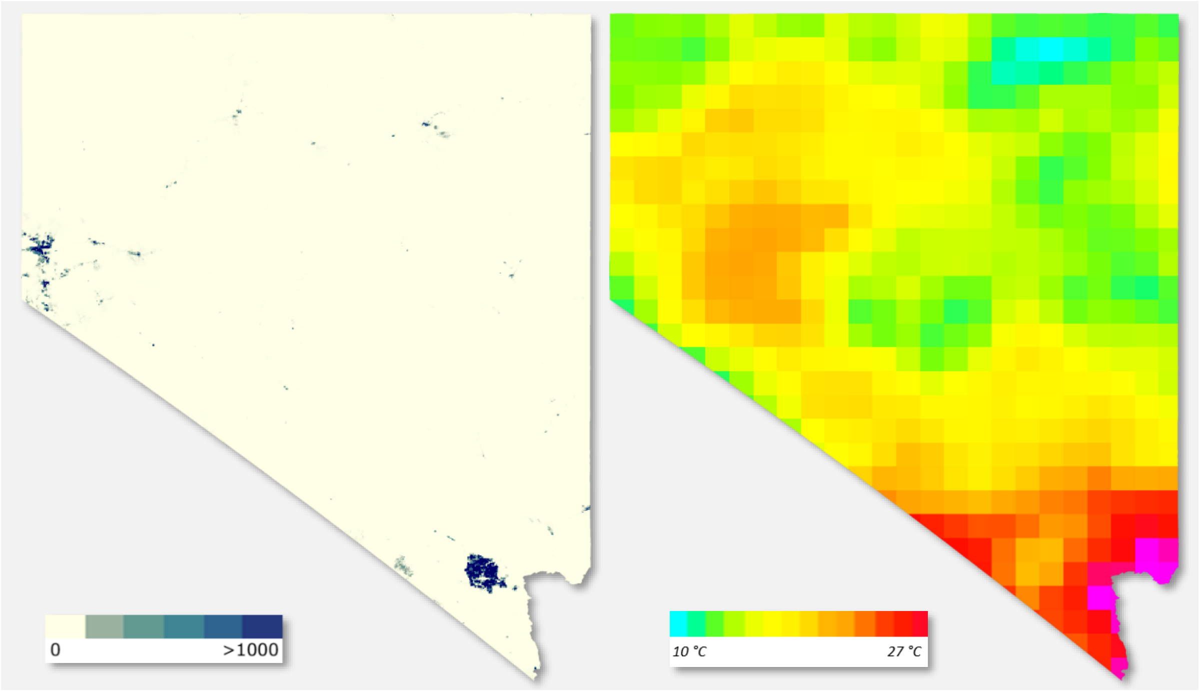

**SUPPLEMENTARY FIGURE S3.** Flowchart of the Filtering Process.

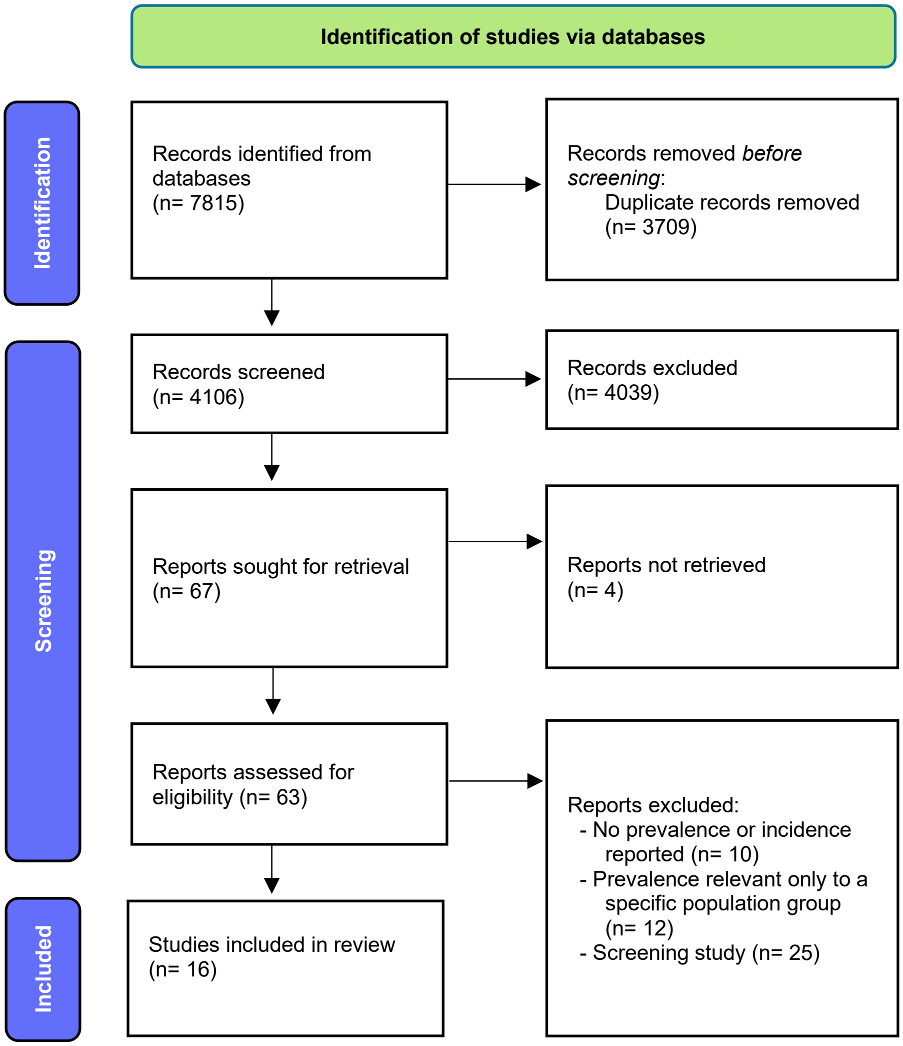

**SUPPLEMENTARY FIGURE S4:** Population-weighted climate variables for every study area, sorted by prevalence.

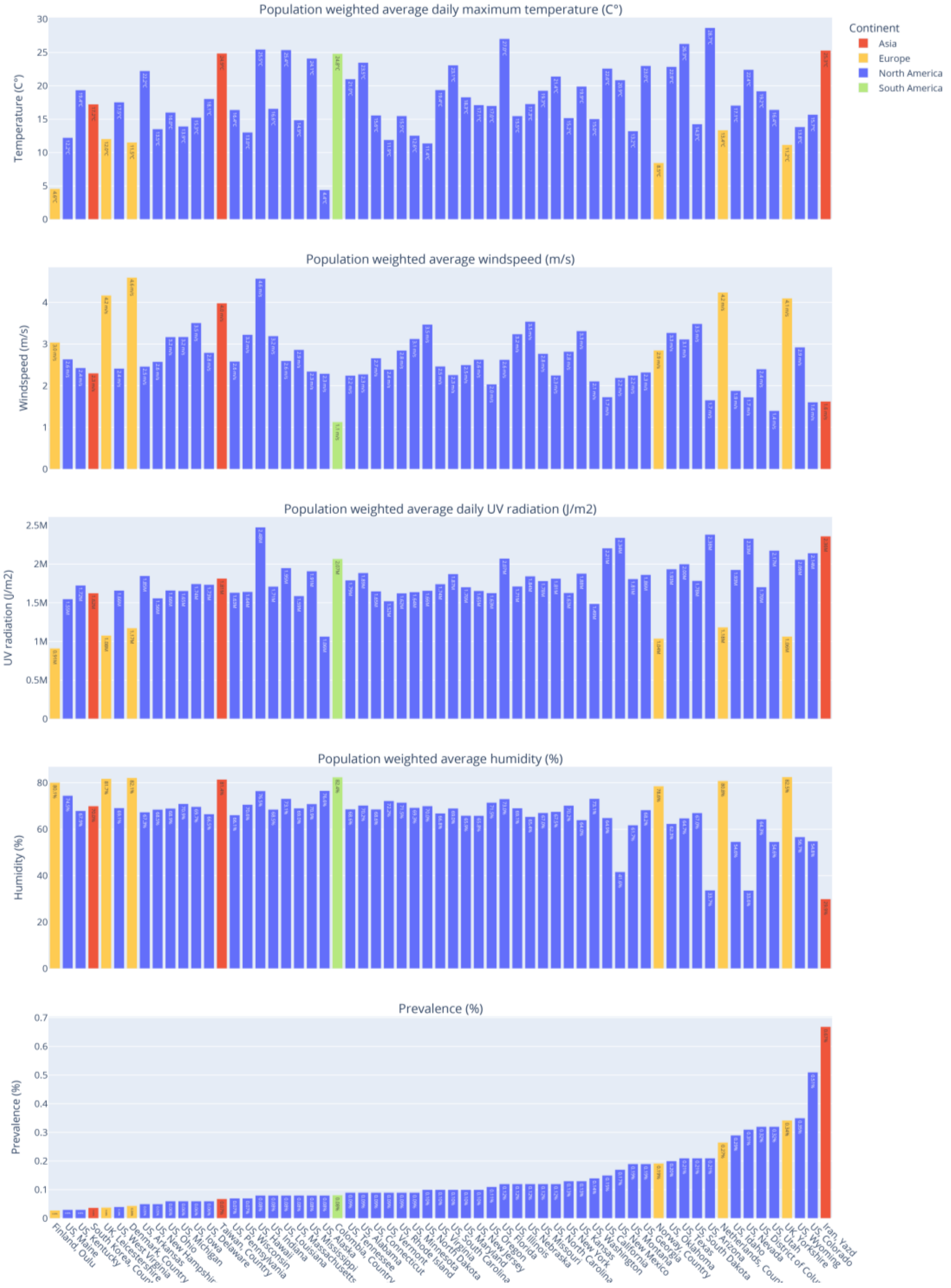

**SUPPLEMENTARY METHODS S1. Detailed Literature Search Strategy**

**Databases Searched**

In order to systematically review the prevalence of keratoconus, a broad range of relevant databases were selected and searched. These databases are listed in the table below:

| Database searched | Platform | Years of coverage |
|---|---|---|
| Medline All | Ovid | 1946 - February 2024 |
| Embase | Embase.com | 1971 - February 2024 |
| Web of Science Core Collection* | Web of Knowledge | 1975 - February 2024 |
| LILACS (Latin America and the Caribbean) | Lilacs.bvsalud.org | 1982 - February 2024 |
| Scientific Electronic Library Online (SciELO) | Scielo.org | 1997 - February 2024 |
| Global Index Medicus | Globalindexmedicus.net | 1879 - February 2024 |

* Science Citation Index Expanded (1975-present); Social Sciences Citation Index (1975-present); Arts & Humanities Citation Index (1975-present); Conference Proceedings Citation Index- Science (1990-present); Conference Proceedings Citation Index- Social Science & Humanities (1990-present); Emerging Sources Citation Index (2005-present).

**Keywords and Keyphrases**

A suitable and comprehensive search strategy was developed for each database with the help of a librarian (W.B.) who specializes in systematic reviews. The following search strategies were followed per database:

**Medline**

(Keratoconus / OR (keratoconus* OR keratoconic* OR ((conic* OR ectasia*) ADJ3 cornea*)).ab,ti,kw.) AND (Epidemiology/ OR Mass Screening/ OR Probability/ OR Population Groups/ OR Population/ OR (epidemiolog* OR prevalen* OR inciden* OR epidemiolog* OR probabilit* OR determinant* OR predict* OR frequenc* OR survey* OR population* OR screening).ab,ti,kw.) NOT (exp animals/ NOT humans/)

**Embase**

(keratoconus/de OR 'cornea ectasia'/de OR (keratoconus* OR keratoconic* OR ((conic* OR ectasia*) NEAR/3 cornea*)):Ab,ti) AND ('epidemiological data'/exp OR epidemiology/exp OR screening/exp OR probability/de OR 'predictive value'/de OR population/de  OR 'population research'/de OR prediction/de OR (epidemiolog* OR prevalen* OR inciden* OR epidemiolog* OR probabilit* OR determinant* OR predict* OR frequenc* OR survey* OR population* OR screening):Ab,ti) NOT ([conference abstract]/lim AND [2000-2020]/py) NOT ([animals]/lim NOT [humans]/lim)

**Web of Science Core Collection**

TS=(((keratoconus* OR keratoconic* OR ((conic* OR ectasia*) NEAR/2 cornea*))) AND ((epidemiolog* OR prevalen* OR inciden* OR epidemiolog* OR probabilit* OR determinant* OR predict* OR frequenc* OR survey* OR population* OR screening)))  NOT DT=(Meeting Abstract OR Meeting Summary) AND LA=(English)

**LILACS, Scielo and Global Index Medicus**

((keratoconus* OR keratoconic* OR ((conic* OR ectasia*) AND cornea*))) AND ((epidemiolog* OR prevalen* OR inciden* OR epidemiolog* OR probabilit* OR determinant* OR predict* OR frequenc* OR survey* OR population* OR screening))

**SUPPLEMENTARY METHODS S2. Prevalence Estimation Method**

This supplement shows how the point prevalence was derived from incidence in studies which reported incidence but did not explicitly report the point prevalence of keratoconus. The following formula was used:

$$Prevalence = Incidence * Duration\ of\ Disease$$

Where:

$$Duration\ of\ Disease = Local\ Average\ Life\ Expectancy - Mean\ Age\ at\ Diagnosis$$

The local average life expectancy at birth for the average-aged patient was deemed the most appropriate number to use in this context. Life expectancy was extracted from the United Nations World Population Prospects report (2022 revision).[1] The relevant reference year was identified as follows:

$$Reference\ Year = Data\ Collection\ Year - Mean\ Age\ at\ Diagnosis$$

Data collection year was the year corresponding to the midpoint of data collection for each study. The reported mean age at diagnosis was used as it is whenever it was reported in the study. In the Study of Mejia-Salgado et al., we calculated it using the incidence table.[2] For Taiwan, calculation was not possible.[3] Our attempt to contact the corresponding author failed, so the mean age at diagnosis from another Taiwanese keratoconus study[4] was used.

| First Author (Publication Year) | Study Area | Yearly Incidence (per 100.000) | Mean Age at Diagnosis | Reference Year | Data Collection Year | Local Average Life expectancy | Duration of Disease | Calculated Point Prevalence |
|---|---|---|---|---|---|---|---|---|
| **Georgiou (2004)[5] [all ethnicities]** | Yorkshire (United Kingdom) | **6.98** | 23.40 | **1974** | 1997 | 72.4 | 49 | 0.34% |
| **Georgiou (2004)[5] [white patients]** | Yorkshire (United Kingdom) | **3.33** | 26.4 | 1974 | 1997 | 72.4 | 46 | 0.15% |

| | | | | | | | | |
|---|---|---|---|---|---|---|---|---|
| **Mejia-Salgado (2023)[2]** | **Colombia** | **2.07** | 30.99 | **1986** | 2017 | 70.2 | 39.21 | 0.08% |
| **Ng (2023)[3]** | **Taiwan** | **1.56** | 29.76 | **1979** | 2009 | 73.5 | 43.74 | 0.07% |
| **Ziaei (2012)[6]** | **Province of Yazd (Iran)** | **22.30** | 28.50 | **1980** | 2008 | 58.5 | 30 | 0.67% |

## SUPPLEMENTARY METHODS S3. Climate Data Extraction and Processing

### Raw Climate Data Extraction

The specific datasets that were used for the extraction of climate variables are: (1) ERA5 Daily Aggregates via Google Earth Engine (ERA5D) for daily wind speed and temperature data,[7] (2) ERA5 monthly averaged data on single levels from 1940 to present (ERA5M) for UV data,[8] and (3) Essential Climate Variables for assessment of climate variability from 1979 to present (ECV) for monthly humidity data.[9] The analysis of the climate parameters was performed using Google Earth Engine (GEE),[10] a cloud-based platform that provides access to (analysis of) a large collection of geospatial data. The ERA5D dataset was already available in GEE by default, ERA5M and ECV had to be downloaded from the C3S Climate Data Store (CDS), converted and uploaded as an asset to GEE.

Daily maximum temperature was chosen over mean temperature to better reflect daytime exposure when individuals are awake. Wind speed was calculated from eastward and southward components in the ERA5D. One day in the ERA5D dataset had erroneous wind speed data, this day was excluded from all wind speed averages. Relative humidity was obtained from monthly ECV dataset. UV radiation data was available in monthly aggregates, but lacked distinction of UVA specifically, which is of particular interest to the studied disease. Severe outliers were excluded from the 10-year averages by visual inspection.

### Uploading Climate Data into Google Earth Engine

UV data and relative humidity data were not readily available in the Google Earth Engine (GEE). They therefore had to be downloaded from the European Centre for Medium-Range Weather Forecasts' (ECMWF) Copernicus Climate Change Service (C3S) Climate Data Store (CDS) and ingested into GEE.

UV data was downloaded from the ERA5 monthly averaged data on single levels from 1940 to present (ERA5M),[8] available at: https://cds.climate.copernicus.eu/cdsapp#!/dataset/reanalysis-era5-single-levels-monthly-means.

Relative humidity was downloaded from Essential Climate Variables (ECV) for assessment of climate variability from 1979 to present,[9] available at: https://cds.climate.copernicus.eu/cdsapp#!/dataset/ecv-for-climate-change?tab=overview.

The strategy for the conversion inspired by Julia Wagemann's scripts on GitHub.[11] To summarize, images were downloaded from the CDS in GRIB format to a local computer. The GRIB files needed to be converted to GeoTIFF in order to be ingested by GEE. This conversion was performed using GDAL library[12] for Python 3.8. For each GRIB a JSON file was created with upload instructions for ingestion in GEE, a so called manifest upload, in order to bulk upload images. The JSON manifest contains information about acquisition date and other properties. After conversion to GeoTIFF, image files were uploaded to Google Cloud Platform (GCP). From GCP, images could be uploaded as assets to GEE in combination with the manifest JSON files.

UV data had erroneous timestamps which were all shifted 6 hours in advance. These timestamps were fixed by adding 6 hours to the timestamp.

**Population Weighting**

Population weighting is especially important in areas with a diverse climate and with varying population density. Population data was acquired from the Gridded Population of the World version 4 (GPWv4) Revision 11 population count dataset.[13] Population count estimates were available for the years: 2000, 2005, 2010, 2015 and 2020. The following formula was used to calculate the population-weighted exposure (*Exp*).

$$Exp = \sum_{i}^{n} \left( \frac{Pi}{P} \cdot X_i \right)$$

*Equation 1*

*n* is the amount of pixels in the area of interest.

*Pi* is the subpopulation in that pixel.

P is the total population in the area of interest.

And *Xi* is the value of the climate parameter at pixel *i*.

A 10-year mean of climate variables was calculated for each study. If two population estimates from different years were included within this period, their mean was utilized. Studies predating 2000 lacked population density estimates, so the 2000 estimate was used instead. For studies predating available climate data (prior to 1989), climate conditions from 1979 to 1989 were used.

**SUPPLEMENTARY REFERENCES**


1. United Nations Population Division. *World Population Prospects 2022*. Available from: https://population.un.org/wpp/Download/Files/5_Archive/WPP2022-Excel-files.zip.
2. Mejia-Salgado G, Cifuentes-González C, Rojas-Carabali W, et al. Colombian Ocular Diseases Epidemiology Study (CODES): incidence and sociodemographic characterisation of keratoconus between 2015 and 2020. *BMJ open ophthalmology* 2023;8:e001238.
3. Ng JM, Lin KK, Lee JS, Chen WM, Hou CH, See LC. Incidence and prevalence of keratoconus in Taiwan during 2000-2018 and their association with the use of corneal topography and tomography. *Eye (Lond)* 2024;38:745-751.
4. Lin KK, Lee JS, Hou CH, et al. The Sociodemographic and Risk Factors for Keratoconus: Nationwide Matched Case-Control Study in Taiwan, 1998-2015. *Am J Ophthalmol* 2021;223:140-148.
5. Georgiou T, Funnell CL, Cassels-Brown A, O'Conor R. Influence of ethnic origin on the incidence of keratoconus and associated atopic disease in Asians and white patients. *Eye* 2004;18:379-383.
6. Ziaei H, Jafarinasab MR, Javadi MA, et al. Epidemiology of Keratoconus in an Iranian Population. *Cornea* 2012;31:1044-1047.
7. Hersbach H, Bell B, Berrisford P, Biavati G, Horányi A, Muñoz SJ, Nicolas J, Peubey C, Radu R, Rozum I, Schepers D, Simmons A, Soci C, Dee D, Thépaut JN. ERA5 hourly data on single levels from 1940 to present. In: Copernicus Climate Change Service (C3S) Climate Data Store (CDS) (ed); 2023.
8. Hersbach H, Bell B, Berrisford P, Biavati G, Horányi A, Muñoz SJ, Nicolas J, Peubey C, Radu R, Rozum I, Schepers D, Simmons A, Soci C, Dee D, Thépaut JN. ERA5 monthly averaged data on single levels from 1940 to present. In: Copernicus Climate Change Service (C3S) Climate Data Store (CDS) (ed); 2023.

9. Hersbach H, Muñoz SJ, Nicolas RI, Simmons VFA, Bell B, Berrisford P, Biavati G, Buontempo C, Horányi AJ, Peubey C, Radu R., Schepers D, Soci C, Dee D, Thépaut JN. Essential climate variables for assessment of climate variability from 1979 to present. In: Copernicus Climate Change Service (C3S) Data Store (CDS) (ed); 2018.

10. Gorelick N, Hancher M, Dixon M, Ilyushchenko S, Thau D, Moore R. Google Earth Engine: Planetary-scale geospatial analysis for everyone. *Remote Sens Environ* 2017;202:18-27.

11. Wagemann J. *ERA5 reanalysis data in Google Earth Engine*: Github; 2021. Available from: https://github.com/jwagemann/era5_in_gee.

12. GDAL/OGR contributors. GDAL/OGR Geospatial Data Abstraction software Library. Open Source Geospatial Foundation; 2024.

13. Center for International Earth Science Information Network CCU. *Gridded Population of the World, Version 4 (GPWv4): Population Count, Revision 11*. Palisades, New York: NASA Socioeconomic Data and Applications Center (SEDAC); 2018.
